# Neurobehavioral Initiation and Motivation Problems After Acquired Brain Injury

**DOI:** 10.3389/fneur.2020.00023

**Published:** 2020-02-21

**Authors:** Simona Palmisano, Luciano Fasotti, Dirk Bertens

**Affiliations:** ^1^Donders Institute for Brain, Cognition and Behaviour, Radboud University, Nijmegen, Netherlands; ^2^Rehabilitation Center Klimmendaal, Arnhem, Netherlands

**Keywords:** initiation, motivation, apathy, adynamia, acquired brain injury

## Abstract

Motivation is a primary and permanent source of human behavior and adaptation. Motivational deficits, along with deficiencies in initiation, frequently occur in individuals with acquired brain injury (ABI). These neurobehavioral problems are associated with consequences at the participation level: patients are reluctant to engage in rehabilitation, and their subsequent social reintegration is often at risk. The same problems may also become a heavy burden for the families of individuals with ABI. In the present paper, we will critically review both the current definitions and the instruments used to measure motivational disorders following ABI. We will also describe the neural system underlying motivation and its impairments. What emerges is the need to develop specific rehabilitative treatments, still absent at the moment, with the ultimate aim of ensuring a better quality of life for both the patients and their proxies.

## Introduction

According to the World Health Organization (1), an acquired brain injury (ABI) is a brain lesion occurring after birth that cannot be related to a congenital or degenerative disease. Distressing physical and cognitive disabilities are well-known consequences of ABI. However, the ensuing changes in neurobehavioral functioning may even be more overwhelming for both the patients as well as their proxies (2).

Neurobehavioral disability (NBD) (3) is a term used to describe neuropsychological disabilities and behavioral disturbances in individuals with ABI (4). The concept was introduced to emphasize the idea that disorders of cognition, social behavior, emotional expression, and personality are connected in persons with ABI and may ultimately result in disrupted and provocative behavior. According to Wood (5), NBD may include executive and attentional impairments; lack of insight and awareness; social judgment problems; labile mood; inadequate impulse control; and several personality changes. These consequences of ABI undermine the capacity for independent social behavior and result in severe long-term social impairment, leading to poor psychosocial outcomes (6). Moreover, they affect not only the survivors of brain injury but also their whole families (2).

Within NBD and motivational deficits, lack of behavioral initiation is a consequence of ABI that proxies often report as the most difficult to deal with (7, 8). Initiation is a crucial aspect of motivation, as it represents the ability to start the execution of a task. But other features of motivation, like the paucity of goal directedness, may also be distressing and clinically significant. It is therefore important to define motivational disorders that afflict ABI survivors more clearly. This may contribute to the development of more adequate diagnostic tools as well as rehabilitation treatments that may lead to better living conditions for both the patients and those surrounding them.

## Motivational Disorders in Acquired Brain Injury Survivors

Motivation contributes to adaptive functioning and is an important determinant of quality of life. It is the process that starts, regulates, and maintains goal-directed behaviors (9). Goal-directed behavior is composed of a set of associated processes (i.e., motivational, cognitive, emotional, and motor) allowing the achievement of a goal, by translating an internal state into action (10, 11). Such a goal might be immediate and physical, or long-term and abstract (11). As stated by Nevid (9): “Motives are the “whys” of behavior, the needs or wants that drive behavior and explain what we do.”

Disorders of diminished motivation (DDM) are characterized by impairments in goal-directed behavior, thought, and emotion (12). These disorders occur frequently in individuals after an ABI: without apparent motivation, these individuals fail to stay on their medication, keep appointments, maintain interactions with their relatives and friends, or resume their jobs.

DDM can be clinically observed as a gross underproduction of speech, movement, and emotional response and include *akinetic mutism, abulia*, and *apathy* (13).

The most disabling condition within DDM is akinetic mutism. Akinetic mutism is characterized by an inability to voluntarily initiate motor or verbal responses, in the presence of preserved arousal and sensorimotor functions (14, 15). It is a severe clinical condition in which the person is totally deprived of motivation, devoid of primary needs, and characterized by a severe reduction of motricity, facial expressions, gestures, and verbal communication. However, these persons still retain some degree of alertness (16, 17).

Abulia, defined by Berrios and Gili (18) as a disorder of the will, is positioned in the middle of the spectrum of DDM. Although individuals with abulia show less severe symptoms than do persons with akinetic mutism, these symptoms are qualitatively identical: passivity, reduced spontaneous behavior and speech, lack of initiative, and psycho-motor slowing, combined with a reduced emotional responsiveness and spontaneity. According to Marin and Wilkosz (12), abulia results into akinetic mutism when it is exacerbated and into apathy when it is improved.

Apathy is a state of overt diminution in motivation, compared with an individual's previous state, although it is not related to cognitive, emotional, or motor deficits (19). It directly involves the person's goal-directed behavior, entailing a reduction of emotional engagement and a difficulty in initiating new actions (20). Marin and Wilkosz (12) purported that apathetic patients are able to start and pursue actions, report their intentions, and show emotional responses to major events. However, these behaviors are not as intense, less extensive, and shorter than in non-apathetic persons.

Levy and Dubois (21) have defined apathy as “the quantitative reduction of self-generated, voluntary and purposeful behaviors.” They have identified three dysfunctional domains in apathetic individuals: the “affective-emotional” domain, in which an individual is incapable to establish a relation between emotional-affective expressions and ongoing or future behavior; the “cognitive” domain, which entails difficulties in devising a plan needed for ongoing or forthcoming behavior; and the “auto-activation” domain, which refers to the inability to activate and initiate thoughts and actions, combined with a relatively adequate skill to generate externally guided behavior. Deficits in auto-activation lead to a disruption in activation (also known as “psychic akinesia” or “athymhormia”) and may be considered the most severe form of apathy (21).

Apathy is among the most common sequelae of ABI. There is no obvious relationship between the brain injury severity and the appearance of apathy. Moreover, apathy is generally unrelated to time since injury and has no significant association with either age at injury or educational level (22).

Prigatano (23) described the psychosocial problems associated with lack of motivation, also termed *amotivation* or *adynamia*, in patients with ABI. Amotivation and adynamia are related to the negative symptoms of apathetic behavior and anhedonia (24). Negative symptoms deal with behaviors, thoughts, or feelings normally present that are diminished or completely absent. It is also common that patients express a lack of motivation by reporting a decreased level of energy (anergia) or an abnormal physical or mental fatigue (24). As a result, these subjects may be seen as passive, apathetic, or depressed because they seem drained and uninterested in their environment. Anhedonia is defined as a consistent and marked reduction of interest or pleasure in previously rewarding activities (25).

Adynamia may result in considerable difficulties with new or more complex activities or behaviors, particularly those consisting of many steps, or entailing a sequence of steps to be achieved (26). So adynamia contributes to problems in many areas of life such as social functioning problems and difficulties in returning to work or study. It also negatively affects the learning of coping strategies and the application of skills trained during rehabilitation. Social isolation is commonly seen as a result of the patients' lack of motivation to interact with their environment (5). However, adynamia does not always means that persons feel unmotivated: although starting or completing a task is difficult, they often talk about their plans, goals, and planned activities. Individuals with adynamia often know what they want to do, but they lack the drive to actually start the activity (26). Some clinicians also use the term avolition for this symptom (24). The American Psychiatric Association (27) defined avolition as “a decrease in the motivation to initiate and perform self-directed purposeful activities.” Hence, people with avolitional disorders encounter difficulties in initiating behaviors, although they can show these behaviors when verbally prompted to do so (24).

In this context, Laplane (28) introduced the concept of “loss of psychic self-activation” (LPSA) to describe a syndrome characterized by an almost complete lack of initiative, a strong reduction in spontaneous motor activity and speech, and an absence of self-initiated mental activity of any kind. A person with LPSA experiences a feeling of “mental emptiness,” an indifference with regard to previous interests, and a flattened affect (29). Strikingly, the absence of self-initiated activity may disappear in reaction to external stimulation (30). Thus, in some cases, verbal reminders and prompts are useful to stimulate individuals with ABI to start activities. However, additional cues are often necessary to stimulate patients to complete a task (26).

## Brain Regions Involved in Motivational Disorders

Several empirical studies have revealed the involvement of subcortical–cortical circuits in the initiation of cognition and behavior. The generation of motivated behavior in healthy people involves a network of medial frontal and striatal regions (31).

**Figure 1:**
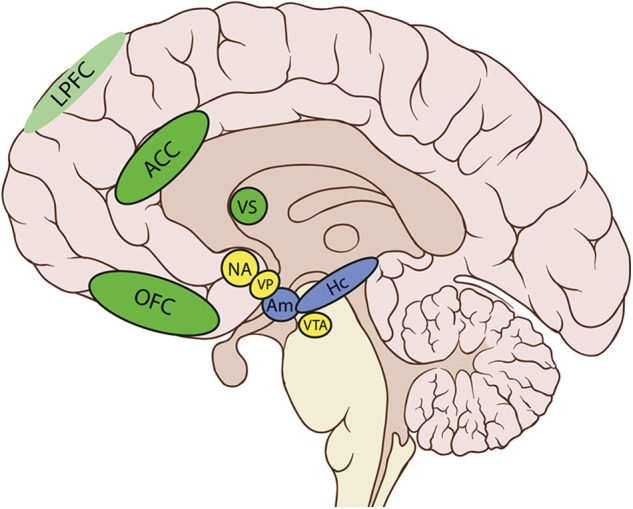
Anatomical areas involved in motivation.

In particular, the cortico-basal ganglia loop involving the ventral striatum (VS) plays a key role in the generation of motivational processes (32–35). The disruption of this loop produces akinetic mutism, abulia, or apathy (12). In this cortico-striatal-pallidal-thalamic circuit, the dorsal parts of the anterior cingulate cortex (ACC) and the orbitofrontal cortex (OFC), the nucleus accumbens (NA), the ventral pallidum (VP), and the ventral tegmental area (VTA) are crucial areas in both the initiation and maintenance of adequate motivational levels [(24), see Table 1 and Figure 1].

**Table 1:** Cortical and subcortical regions and their putative contribution to motivational processes.

**Cortical**	**Subcortical**	**Process**
	- Amygdala (Am) - Hippocampus (Hc)	- Collect internal and external information (motivational input)
- dorsal Anterior Cingulate Cortex(dACC) - Orbitofrontal Cortex (OFC) - lateral Prefrontal Cortex (lPFC)	- Ventral Striatum (VS)	- Assess and motivate choices leading to effort - Update the value of choices
	- Nucleus Accumbens (NA) - Ventral Pallidum (VP) - Ventral Tegmental Area (VTA)	- VTA + medial NA-VP: receive limbic input from Am and Hi - VTA + ventral NA-VP: transmit to motor output systems (motor cortex, basal ganglia,…)

The involvement of some of these areas in motivated behavior has been confirmed by neuroimaging studies. These studies have shown that atrophy or functional disruption of the medial frontal cortex—in particular the dorsal ACC (dACC) and the OFC—are significantly related to apathy. Moreover, damage in subcortical areas such as the VS, the medial thalamus, and the VTA may also lead to apathy. Finally, disruption of the connections between all these regions contributes to apathy as well (24, 31). These brain–behavior relations have been established with several imaging techniques, including metabolic imaging methods. Gray matter (GM) atrophy findings and both structural and functional connectivity studies have confirmed these associations (31). The ACC and the VS seem to play an essential role in assessing and motivating choices that will lead to effort, and also in supporting the motivation required to sustain behavior until the attainment of a goal. Aversion to effort due to alterations in response within the ACC and VS may result in lack of motivation and therefore apathy (31).

The NA and the VP have more medial and lateral areas, which are connected to other different brain regions. Medial portions receive limbic input from the amygdala and the hippocampus, necessary to modify the current motivational state (32). The amygdala and hippocampus, as well as the prefrontal cortex (PFC), collect information from the current environment and the drive state of the organism, so as to modulate information in the circuit. In fact, neurons in these regions allow to record changes in the reward significance of the environment, and this could explain why damage to these brain structures presents as apathy (12). The involvement of the PFC in the occurrence of apathy after ABI has also been confirmed in group studies of patients with lesions in this area. In these studies, typical behavioral changes, such as impairments of goal-directed behavior and blunted affect, have been identified (36). In particular, the ventromedial PFC (vmPFC), including the OFC, has been mainly associated with valuation, reward learning, emotional regulation, and decision making, whereas the lateral PFC has a key role in executive control or the ability to synchronize thoughts and actions with internal goals, a process leading to effort (11). Paradiso et al. (37) even found that individuals with lateral prefrontal damage showed more symptoms of apathy than those with medial frontal damage, suggesting that damage to this area may also severely disrupt motivation. However, the ability to feel and report negative emotions was intact in these patients. Apathy in the traumatic brain injury (TBI) population may also be due to the dysfunction of another cortical area, the insula (11). The anterior insula, through its connections with the amygdala, hippocampus, ACC, and OFC, computes higher-order meta-representations of the primary interoceptive activity. This activity is related to the feeling of pain and its emotional awareness (38). Therefore, damage to insular areas may result in decreased motivation, due to an absence of awareness of emotional and motivational feelings (11).

Through the motor cortex, the reticulospinal tract, the pedunculopontine nucleus, and the basal ganglia (BG), the lateral portions of NA and VP, are connected to output circuits. The BG are involved in many aspects of goal-directed behavior, including the control of movement, and also in mechanisms that drive actions, such as cognition, emotions, and motivation (39). BG are probably a crucial network underlying motivational processes, whereby expected rewards trigger the occurrence of behavior without requiring the persons' awareness (11).

The involvement of the above-described circuit in the occurrence of apathy after ABI has also been confirmed by a study in which event-related potentials (ERPs) were used to investigate the neuronal mechanisms underlying apathy (40). As expected, the authors found changes in the amplitude of the novelty P3 wave, correlated with apathy severity and occasioned by disturbances in the fronto-subcortical circuit.

Levy and Dubois (21) identified several clinical phenotypes of apathy and speculated that different parts of the segregated PFC–BG circuitry may represent the substrate of these phenotypes. The authors link “emotional affective apathy” with damage to the orbitomedial PFC and the VS. Moreover, they associate “cognitive apathy” with a defective functioning of the lateral PFC areas and the dorsal caudate nuclei. Finally, they hypothesize that a deficit of “auto-activation” may be associated with bilateral lesions of the internal parts of the globus pallidus, bilateral paramedian thalamic lesions, or damage to the dorsomedial PFC.

LPSA has more often been explained by a disruption of the frontal–subcortical circuit that underlies motivation (21, 41), including bilateral lesions of the BG, mainly affecting the caudate, pallidus, and putamen (42, 43).

On a more severe level, abulia may result from the disruption of the neural network involved in task initiation, which incorporates the ACC, bilateral anterior insulae, and the bilateral anterior thalami (14, 44). Akinetic mutism has been found to be associated with lesions to of the AC, either unilaterally or bilaterally (45).

## Role Of Dopamine and Norepinephrine in Motivational Disorders

The pathogenesis of behavioral motivation problems after ABI may also be explained by a neurochemical disruption of the motivational circuitry. Dopamine (DA) seems to be the major neurotransmitter linked to motivation (24). Disorders of the mesolimbic DA system may reduce the capacity of stimuli to activate motivated behavior on hedonic bases, to poor activation and defective directional aspects of motivation for the initiation and constancy of behavior, and to an erroneous learning and evaluation of the costs and benefits of actions (46). DA activity, especially in the striatum, plays a central role in “reward, novelty seeking and response to unexpected events” (12). A reduced synthesis of DA attenuates sensitivity to rewards during decision making (24), whereas increasing levels of DA stimulate incentivization by rewards, and also the readiness to go beyond effort costs (31). Therefore, dysfunction of the mesolimbic and neostriatal DA projection systems may provoke impairments in reward-based decision processes. These processes regulate the motivational load that sustains frontal cognitive processes involved in determining goal-directed behavior (47). All these studies emphasize that motivation strongly relies on dopaminergic activity, which often appears to be affected in ABI (12).

Clinically, DA-based medication has been used in the treatment of a wide range of motivational disorder in patients with TBI (48–50). Anecdotal reports seem to show the benefits of these drugs, but according to Worthington and Wood (22), better-quality trials are needed to support these effects.

Beside DA, norepinephrine may also play a crucial role in the generation of adequate levels of motivation. The so-called noradrenergic system is an important regulator of arousal, and adequate levels of motivation are dependent on appropriate levels of arousal (51). Norepinephrine is mainly released by the locus coeruleus in the brainstem and projects throughout the brain. It affects brain functioning in several ways, by enhancing the processing of sensory stimuli, elevating attentional levels, intensifying the formation of memories, and reinforcing the tendency of the brain to respond to external and internal stimulation. These processes act as prerequisites for the adequate regulation of motivational levels and the initiation of behavior.

Another neurotransmitter linked to motivation is serotonin (24). Depletion of this neurotransmitter changes the attitude of people toward rewards and punishments, whereas administration of a serotonin reuptake inhibitor can influence decision making.

## Assessment of Disorders of Diminished Motivation

As suggested by Spiegel et al. (13), the assessment of patients with diminished motivation should be structured, consider input from both patient and caregiver, and also include the physician's opinion. It should include a complete and systematic neuropsychiatric evaluation, including a picture of the patient's social and physical environment. It is important to investigate the psychosocial history to determine the patient's premorbid levels of motivation and coping skills and to take into account external factors like personal experience or education (12). It is also useful to obtain reports from multiple informants, including both the patient and significant others (11), as some studies have shown that apathetic patients report more severe apathy than do their relatives (52, 53).

To quantify the loss of motivation, several rating instruments have been developed. In a review, Clarke et al. (54) discussed 15 apathy scales or subscales and recommended the “Apathy Evaluation Scale” (AES) and the “Neuropsychiatric Inventory” (NPI) as the most psychometrically robust.

The AES (55) is probably the most widely used assessment instrument. It consists of 18 items and can fill in as a self-rating scale, as caregiver paper-and-pencil test, and a semistructured inventory completed by the clinician (12). The NPI is also extensively used as a valid and reliable instrument. It consists of an interview, administered to the patient's caregiver, and is intended to identify the existence and the severity of 10 non-cognitive symptoms, including apathy (12, 13, 56).

More recently, Ang et al. (57) have introduced the Apathy-Motivation Index (AMI), a reliable short self-report scale designed for assessing motivation and measuring individual levels of apathy. The AMI is a useful instrument to survey different processes underlying deficiencies of motivation in otherwise healthy people. This scale uncovers associations between apathy and comorbid problems in different emotional, social, and behavioral domains.

Alterations in motivation can also be assessed by examining a patient's reactivity to internal or external stimulation (58). The need to design more objective tools to evaluate apathy has led Muller et al. (52) to log everyday motor activity in patients with acquired brain damage. The extent of apathy is assessed by measuring the rate of self-initiated behavior. This type of instrument allows to relate the signs of apathy to the performance in other behavioral and cognitive tasks. Examples of behavioral tasks include gambling or reversal tasks investigating the ability to adapt behavior in function of expected rewards. The Wisconsin Card Sorting Test, the Tower of London test, or fluency tests are examples of useful instruments to establish a relation between apathy and cognitive inertia (59).

## Rehabilitative Interventions

Given the frequency of severe motivational symptoms in patients with ABI and the problems they bring about in terms of loss of social participation, economic and occupational cost, and especially caregivers' well-being, it seems extremely important to develop adequate rehabilitation interventions to alleviate these personal and social costs and to ensure a better quality of life for both the patients and their proxies.

Unfortunately, specific treatments for initiation and motivation problems after ABI are rare and often not evaluated in well-designed studies. In most of the cases, psychological treatments are not specifically designed for initiation or motivation problems, and they generally incorporate a variety of specific cognitive rehabilitative techniques, or behavioral modification methods, or both (12, 60, 61). Cognitive rehabilitation therapies utilize techniques found in problem-solving therapy, based on strategies to improve goal-directed behavior by teaching better planning, execution, and monitoring of activities (61). Other cognitive interventions use external compensation strategies like checklists and paging systems to stimulate initiation toward goal-directed activities (62).

Examples of behavioral therapies are activity therapy (63), multi-sensory stimulation (64), and music therapy (65). These therapies have been shown to diminish apathy to some extent in neurological populations with progressive disorders, in particular Alzheimer's dementia. However, a majority of these studies lack rigorous designs for unbiased evaluation of treatment effects. Therefore, the obtained results may actually be due to factors such as spontaneous recovery of apathy, rater expectations of gains, or non-specific effects, given the frequent lack of a control group. Another widely used behavioral technique is goal-setting therapy (61), which consists in using goals to provide targets for patients to work toward (66). Goal-setting therapy is based on the idea that explicit goals trigger action (67) and that conscious human behavior is directed and driven by individual goals. The technique allows targeting of individual goals and effects to be readily measured (68). To this date, only one study (69) used goal setting in a neurological population. In a sample of 100 patients, 78% of the long-term goals set by the participants were achieved, indicating that goal-directed activity was successfully accomplished. On the other hand, in a study with brain injury subjects comparing cognitive behavioral therapy (CBT) and a peer support group (70), no significant improvements in functioning were found for either group on the subscales “executive dysfunction” and “apathy” of the Frontal Systems Behavior Scale. It is clear that further studies, specifically investigating the effectiveness of apathy treatment in individuals with ABI (of non-progressive nature), are needed.

In apathy, communicative and cognitive skills are often preserved, and therefore, psychological and social interventions are the treatments of choice. On the other hand, the treatment of more severe disorders like akinetic mutism and abulia is mainly pharmacological (12). Pharmacological interventions are often based on the prescription of DA agonists (71). Several studies suggest that the use of acetylcholinesterase inhibitors and psychostimulants may also be effective in the pharmacological improvement of apathy (61, 72).

Although specific treatments are scarce, some general recommendations concerning rehabilitation of apathy have been made. First of all, it is indispensable to optimize the patient's general medical condition, which contributes to positive effects on motivation (12). The improvement of general physical condition can enhance functional skills, energy, and drive, thus increasing the patient's expectation that taking initiatives and sustaining efforts may lead to the attainment of behavioral goals.

The treatment of neurobehavioral motivation problems after ABI should be based on thorough assessment, followed by an estimation of a patient's losses and residual capacities (73). This allows the design of “psycho-prostheses” that enable patients to compensate for their deficits and help them to make the best possible use of their residual capacities (12).

Target behaviors and baseline frequencies should be identified prior to treatment (73), and therapeutic goals should be established in collaboration with the patient, to reinforce engagement and intensify the patient's feeling of control and belief in success (12). It is important to make use of personalized treatments (24) —pharmacological or psychological—and to also pay attention to the physical and psychological determinants of apathy (73).

Other important variables contributing to effective treatment are the modification of the patient's environment and the participation of family members and professional therapists in the treatment of DDM (12). The objective of environmental interventions is to strengthen the rewarding potential of the environment, by introducing new of familiar sources of interest, pleasure, stimulation, and also socialization. Finally, psycho-education, professional counseling, and psychotherapy interventions should not be overlooked, as they may help in dealing with injury-related losses, interpersonal problems, or family stressors related to individual determinants of initiation and motivation problems (12).

However, methodologically more rigorous studies have to be designed and performed in order to investigate the effectiveness of different treatment techniques aimed at improving initiation and motivation problems after ABI. In particular, more randomized controlled trials comparing the different ways of addressing apathy are required. These trials should be conducted with larger sample sizes than those of the studies already carried out. Furthermore, the use of more sophisticated research designs and appropriate statistical analyses are needed to examine both the effects of therapies and the differences between groups of patients with distinct types of brain injuries. In order to compare treatments and their implementability, a more standardized terminology and better operationalized definitions of motivation and initiation disorders are also required.

## Conclusions

Motivation is a ubiquitous and crucial determinant of behavior and adjustment. Deficits in self-initiated, goal-directed motivated behavior are common after ABI, representing one of the most draining legacies of the injury for the patient and for his/her proxies. These deficits seem to be related to malfunctioning of DA activity and to dysfunction of a network of medial frontal and striatal regions. Current knowledge of the normal function of these brain areas in motivated behavior allows straightforward and hypothesis testing approach to DDM, with predictions that can be verified.

Although some promising tools for assessing apathy are currently available, in the field of treatment, an unsatisfactory and worrying situation emerges. For the time being, there are only generic recommendations but no evidence-based specific interventions that support a targeted treatment of initiation and motivation problems for patients with ABI.

The goal of future research should be to better define and operationalize the constructs of motivation and initiation disorders. These may contribute to design increasingly valid assessment tools, with the ultimate aim to develop effective and personalized treatments for patients suffering from these disabling symptoms. By improving treatments, it will be possible to offer persons with ABI a way to improve their functional capacities and thus to ensure a better quality of life for both the patients and their proxies.

## Author Contributions

All authors listed have made a substantial, direct and intellectual contribution to the work, and approved it for publication.

### Conflict of Interest

The authors declare that the research was conducted in the absence of any commercial or financial relationships that could be construed as a potential conflict of interest.

## Glossary

- Abulia: a less severe type of apathy than akinetik mutism, characterized by lack of initiative, passivity and a reduction of verbal and motor responses.
- Adynamia: a decrease of vital power that leads to a lack of behaviour, thought and feeling normally present.
- Akinetic mutism: the most severe form of apathy, in which a person does not show motor or verbal responses anymore, despite a relatively preserved alertness.
- Avolition: a lack of motivation necessary to start and accomplish purposeful tasks.
- Apathy: a reduction of self-generated, purposeful behavior.
- Anhedonia: a marked decline of pleasure in activities that were previously rewarding.
- Loss of Psychic Self-Activation (LPSA): a striking loss of self-initiated behaviours coupled with a feeling of mental emptiness, but with normal reactivity to external stimulation.
- Motivation: the needs and wants that fuel behaviour and explain why a person behaves in a certain way.
- Neurobehavioural Disabilities (NBD): a mixture of disorders of cognition, social behaviour, emotional expression and personality that may result in disrupted and provocative behaviour after Acquired Brain Injury.
